# New species and records of *Diaporthe* from Jiangxi Province, China

**DOI:** 10.3897/mycokeys.77.59999

**Published:** 2021-01-14

**Authors:** Qin Yang, Ning Jiang, Cheng-Ming Tian

**Affiliations:** 1 The Key Laboratory for Silviculture and Conservation of the Ministry of Education, Beijing Forestry University, Beijing 100083, China; 2 Forestry Biotechnology Hunan Key Laboratories, Central South University of Forestry and Technology, Changsha 410004, China; 3 The Key Laboratory for Non-Wood Forest Cultivation and Conservation of the Ministry of Education, Central South University of Forestry and Technology, Changsha 410004, China

**Keywords:** DNA phylogeny, five new taxa, forest trees, systematics, taxonomy

## Abstract

*Diaporthe* species have often been reported as important plant pathogens, saprobes and endophytes on a wide range of plant hosts. Although several *Diaporthe* species have been recorded, little is known about species able to infect forest trees in Jiangxi Province. Hence, extensive surveys were recently conducted in Jiangxi Province, China. A total of 24 isolates were identified and analysed using comparisons of DNA sequence data for the nuclear ribosomal internal transcribed spacer (ITS), calmodulin (*cal*), histone H3 (*his3*), partial translation elongation factor-1α (*tef1*) and β-tubulin (*tub2*) gene regions, as well as their morphological features. Results revealed five novel taxa, *D. bauhiniae*, *D. ganzhouensis*, *D. schimae*, *D. verniciicola*, *D. xunwuensis* spp. nov. and three known species, *D. apiculatum*, *D. citri* and *D. multigutullata*.

## Introduction

The genus *Diaporthe* Nitschke (Sordariomycetes, Diaporthales) represents a cosmopolitan group of fungi occupying diverse ecological behaviour as plant pathogens, endophytes and saprobes (Muralli et al. 2006; Rossman et al. 2007; Udayanga et al. 2014, 2015; Fan et al. 2015, 2018; Guarnaccia and Crous 2017; Guarnaccia et al. 2018; Yang et al. 2018, 2020; Manawasinghe et al. 2019; Marin-Felix et al. 2019). *Diaporthe* species are responsible for diseases on a wide range of plant hosts, including agricultural crops, forest trees and ornamentals, some of which are economically important. Several symptoms, such as root and fruit rots, dieback, stem cankers, leaf spots, leaf and pod blights and seed decay are caused by *Diaporthe* spp. (Uecker 1988; Rehner and Uecker 1994; Mostert et al. 2001; Santos et al. 2011; Thompson et al. 2011; Udayanga et al. 2011).

*Diaporthe* was historically considered as monophyletic, based on its typical sexual morph and *Phomopsis* asexual morph (Gomes et al. 2013). However, Gao et al. (2017) recently revealed its paraphyletic nature, showing that *Mazzantia* (Wehmeyer 1926), *Ophiodiaporthe* (Fu et al. 2013), *Pustulomyces* (Dai et al. 2014), *Phaeocytostroma* and *Stenocarpella* (Lamprecht et al. 2011) are embedded in *Diaporthe* s. lat. Furthermore, Senanayake et al. (2017) recently included additional two genera in *Diaporthe* s. lat., namely *Paradiaporthe* and *Chiangraiomyces*.

Species identification criteria in *Diaporthe* were originally based on host association, morphology and culture characteristics (Mostert et al. 2001; Santos and Phillips 2009; Udayanga et al. 2011), which led to the description of over 200 species (Hyde et al. 2020). Some species of *Diaporthe* were reported to colonise a single host plant, while other species were found to be associated with different host plants (Santos and Phillips 2009; Diogo et al. 2010; Santos et al. 2011; Gomes et al. 2013). In addition, considerable variability of the phenotypic characters was found to be present within a species (Rehner and Uecker 1994; Mostert et al. 2001; Santos et al. 2010; Udayanga et al. 2011). During the past decade, a polyphasic approach, based on multi-locus DNA data, morphology and ecology, has been employed for species boundaries in the genus *Diaporthe* (Crous et al. 2012; Huang et al. 2015; Gao et al. 2016, 2017; Guarnaccia and Crous 2017; Guarnaccia et al. 2018; Yang et al. 2018, 2020). The classification of *Diaporthe* has been progressing and the basis for the species identification is a combination of morphological, cultural, phytopathological and phylogenetical analyses (Gomes et al. 2013; Udayanga et al. 2014, 2015; Fan et al. 2015; Huang et al. 2015; Gao et al. 2016, 2017; Guarnaccia and Crous 2017; Guarnaccia et al. 2018; Yang et al. 2018, 2020; Manawasinghe et al. 2019).

In Jiangxi Province, China, some forest trees were observed to be infected with fungal pathogens that cause dieback and leaf spots. Cankered branches and leaves with typical *Diaporthe* fruiting bodies were also found in the area. However, we found that only limited research had been undertaken regarding the fungal pathogens isolated from forest trees in Jiangxi Province. Hence, the present study was conducted to identify *Diaporthe* species that cause dieback and leaf spots disease in the forest trees in Jiangxi Province through morphological and multi-locus phylogenetic analyses, based on modern taxonomic concepts.

## Materials and methods

### Isolates

Fresh specimens of *Diaporthe* were isolated from the collected branches and leaves of six host plants during the collection trips conducted in Jiangxi Province (Table 1). A total of 24 isolates were established by removing a mucoid conidia mass from conidiomata, spreading the suspension on the surface of 1.8% potato dextrose agar (PDA) and incubating at 25 °C for up to 24 h. A single germinating conidium was plated on to fresh PDA plates. Specimens were deposited at the Museum of the Beijing Forestry University (**BJFC**). Axenic cultures were maintained at the China Forestry Culture Collection Centre (**CFCC**).

**Table 1.** Reference sequences included in molecular phylogenetic analyses of *Diaporthe*.

Species	Isolate	Host	Location	GenBank accession numbers				
				ITS	*cal*	*his3*	*tef1*	*tub2*
*D. acericola*	MFLUCC 17-0956	*Acer negundo*	Italy	KY964224	KY964137	NA	KY964180	KY964074
*D. acerigena*	CFCC 52554	*Acer tataricum*	China	MH121489	MH121413	MH121449	MH121531	NA
*D. acutispora*	CGMCC 3.18285	*Coffea* sp.	China	KX986764	KX999274	NA	KX999155	KX999195
*D. alangii*	CFCC 52556	*Alangium kurzii*	China	MH121491	MH121415	MH121451	MH121533	MH121573
*D. alnea*	CBS 146.46	*Alnus* sp.	Netherlands	KC343008	KC343250	KC343492	KC343734	KC343976
*D. ampelina*	STEU2660	*Vitis vinifera*	France	AF230751	AY745026	NA	AY745056	JX275452
*D. amygdali*	CBS 126679	*Prunus dulcis*	Portugal	KC343022	KC343264	KC343506	AY343748	KC343990
*D. angelicae*	CBS 111592	*Heracleum sphondylium*	Austria	KC343027	KC343269	KC343511	KC343753	KC343995
***D. apiculatum***	CGMCC 3.17533	*Camellia sinensis*	China	KP267896	NA	NA	KP267970	KP293476
	**CFCC 53068**	***Rhus chinensis***	**China**	**MK432651**	**MK442973**	**MK442998**	**MK578127**	**MK578054**
	**CFCC 53069**	***Rhus chinensis***	**China**	**MK432652**	**MK442974**	**MK442999**	**MK578128**	**MK578055**
	**CFCC 53070**	***Rhus chinensis***	**China**	**MK432653**	**MK442975**	**MK443000**	**MK578129**	**MK578056**
*D. arctii*	CBS 139280	*Arctium lappa*	Austria	KJ590736	KJ612133	KJ659218	KJ590776	KJ610891
*D. arecae*	CBS 161.64	*Areca catechu*	India	KC343032	KC343274	KC343516	KC343758	KC344000
*D. arengae*	CBS 114979	*Arenga enngleri*	Hong Kong	KC343034	KC343276	KC343518	KC343760	KC344002
*D. aseana*	MFLUCC 12-0299a	Unknown dead leaf	Thailand	KT459414	KT459464	NA	KT459448	KT459432
***D. bauhiniae***	**CFCC 53071**	***Bauhinia purpurea***	**China**	**MK432648**	**MK442970**	**MK442995**	**MK578124**	**MK578051**
	**CFCC 53072**		**China**	**MK432649**	**MK442971**	**MK442996**	**MK578125**	**MK578052**
	**CFCC 53073**		**China**	**MK432650**	**MK442972**	**MK442997**	**MK578126**	**MK578053**
*D. beilharziae*	BRIP 54792	*Indigofera australis*	Australia	JX862529	NA	NA	JX862535	KF170921
*D. betulicola*	CFCC 51128	*Betula albo-sinensis*	China	KX024653	KX024659	KX024661	KX024655	KX024657
*D. biconispora*	CGMCC 3.17252	*Citrus grandis*	China	KJ490597	KJ490539	KJ490539	KJ490476	KJ490418
*D. biguttulata*	CGMCC 3.17248	*Citrus limon*	China	KJ490582	NA	KJ490524	KJ490461	KJ490403
	CFCC 52584	*Juglans regia*	China	MH121519	MH121437	MH121477	MH121561	MH121598
*D. bohemiae*	CPC 28222	*Vitis vinifera*	Czech Republic	MG281015	MG281710	MG281361	MG281536	MG281188
*D. brasiliensis*	CBS 133183	*Aspidosperma tomentosum*	Brazil	KC343042	KC343284	KC343526	KC343768	KC344010
*D. caatingaensis*	CBS 141542	*Tacinga inamoena*	Brazil	KY085927	NA	NA	KY115603	KY115600
*D. caryae*	CFCC 52563	*Carya illinoensis*	China	MH121498	MH121422	MH121458	MH121540	MH121580
*D. celeris*	CPC 28262	*Vitis vinifera*	Czech Republic	MG281017	MG281712	MG281363	MG281538	MG281190
*D. celastrina*	CBS 139.27	*Celastrus* sp.	USA	KC343047	KC343289	KC343531	KC343773	KC344015
*D. cercidis*	CFCC 52565	*Cercis chinensis*	China	MH121500	MH121424	MH121460	MH121542	MH121582
*D. charlesworthii*	BRIP 54884m	*Rapistrum rugostrum*	Australia	KJ197288	NA	NA	KJ197250	KJ197268
*D. cinnamomi*	CFCC 52569	*Cinnamomum* sp.	China	MH121504	NA	MH121464	MH121546	MH121586
***D. citri***	AR 3405	*Citrus* sp.	USA	KC843311	KC843157	NA	KC843071	KC843187
	**CFCC 53079**	***Citrus sinensis***	**China**	**MK573940**	**MK574579**	**MK574595**	**MK574615**	**MK574635**
	**CFCC 53080**	***Citrus sinensis***	**China**	**MK573941**	**MK574580**	**MK574596**	**MK574616**	**MK574636**
	**CFCC 53081**	***Citrus sinensis***	**China**	**MK573942**	**MK574581**	**MK574597**	**MK574617**	**MK574637**
	**CFCC 53082**	***Citrus sinensis***	**China**	**MK573943**	**MK574582**	**MK574598**	**MK574618**	**MK574638**
*D. citriasiana*	CGMCC 3.15224	*Citrus unshiu*	China	JQ954645	KC357491	KJ490515	JQ954663	KC357459
*D. citrichinensis*	CGMCC 3.15225	*Citrus* sp.	China	JQ954648	KC357494	NA	JQ954666	NA
*D. collariana*	MFLU 17-2770	*Magnolia champaca*	Thailand	MG806115	MG783042	NA	MG783040	MG783041
*D. conica*	CFCC 52571	*Alangium chinense*	China	MH121506	MH121428	MH121466	MH121548	MH121588
*D. cucurbitae*	CBS 136.25	*Arctium* sp.	Unknown	KC343031	KC343273	KC343515	KC343757	KC343999
*D. cuppatea*	CBS 117499	*Aspalathus linearis*	South Africa	KC343057	KC343299	KC343541	KC343783	KC344025
*D. discoidispora*	ZJUD89	*Citrus unshiu*	China	KJ490624	NA	KJ490566	KJ490503	KJ490445
*D. endophytica*	CBS 133811	*Schinus terebinthifolius*	Brazil	KC343065	KC343307	KC343549	KC343791	KC343065
*D. eres*	AR5193	*Ulmus* sp.	Germany	KJ210529	KJ434999	KJ420850	KJ210550	KJ420799
*D. fraxini-angustifoliae*	BRIP 54781	*Fraxinus angustifolia*	Australia	JX862528	NA	NA	JX862534	KF170920
*D. fraxinicola*	CFCC 52582	*Fraxinus chinensis*	China	MH121517	MH121435	NA	MH121559	NA
*D. fructicola*	MAFF 246408	*Passiflora edulis* × P. edulis f. flavicarpa	Japan	LC342734	LC342738	LC342737	LC342735	LC342736
*D. fukushii*	MAFF 625034	*Pyrus pyrifolia*	Japan	JQ807469	NA	NA	JQ807418	NA
*D. fusicola*	CGMCC 3.17087	*Lithocarpus glabra*	China	KF576281	KF576233	NA	KF576256	KF576305
*D. ganjae*	CBS 180.91	*Cannabis sativa*	USA	KC343112	KC343354	KC343596	KC343838	KC344080
***D. ganzhouensis***	**CFCC 53087**	**Unknown dead wood**	**China**	**MK432665**	**MK442985**	**MK443010**	**MK578139**	**MK578065**
	**CFCC 53088**	**Unknown dead wood**	**China**	**MK432666**	**MK442986**	**MK443011**	**MK578140**	**MK578066**
*D. garethjonesii*	MFLUCC 12-0542a	Unknown dead leaf	Thailand	KT459423	KT459470	NA	KT459457	KT459441
*D. guangxiensis*	JZB320094	*Vitis vinifera*	China	MK335772	MK736727	NA	MK523566	MK500168
*D. gulyae*	BRIP 54025	*Helianthus annuus*	Australia	JF431299	NA	NA	KJ197271	JN645803
*D. helicis*	AR5211	*Hedera helix*	France	KJ210538	KJ435043	KJ420875	KJ210559	KJ420828
*D. heterophyllae*	CBS 143769	*Acacia heterohpylla*	France	MG600222	MG600218	MG600220	MG600224	MG600226
*D. hispaniae*	CPC 30321	*Vitis vinifera*	Spain	MG281123	MG281820	MG281471	MG281644	MG281296
*D. hubeiensis*	JZB320123	*Vitis vinifera*	China	MK335809	MK500235	NA	MK523570	MK500148
*D. incompleta*	CGMCC 3.18288	*Camellia sinensis*	China	KX986794	KX999289	KX999265	KX999186	KX999226
*D. infecunda*	CBS 133812	*Schinus terebinthifolius*	Brazil	KC343126	KC343368	KC343610	KC343852	KC344094
*D. juglandicola*	CFCC 51134	*Juglans mandshurica*	China	KU985101	KX024616	KX024622	KX024628	KX024634
*D. kadsurae*	CFCC 52586	*Kadsura longipedunculata*	China	MH121521	MH121439	MH121479	MH121563	MH121600
*D. kochmanii*	BRIP 54033	*Helianthus annuus*	Australia	JF431295	NA	NA	JN645809	NA
*D. kongii*	BRIP 54031	*Portulaca grandiflora*	Australia	JF431301	NA	NA	JN645797	KJ197272
*D. litchicola*	BRIP 54900	*Litchi chinensis*	Australia	JX862533	NA	NA	JX862539	KF170925
*D. lithocarpus*	CGMCC 3.15175	*Lithocarpus glabra*	China	KC153104	KF576235	NA	KC153095	KF576311
*D. lonicerae*	MFLUCC 17-0963	*Lonicera* sp.	Italy	KY964190	KY964116	NA	KY964146	KY964073
*D. lusitanicae*	CBS 123212	*Foeniculum vulgare*	Portugal	KC343136	KC343378	KC343620	KC343862	KC344104
*D. masirevicii*	BRIP 57892a	*Helianthus annuus*	Australia	KJ197277	NA	NA	KJ197239	KJ197257
*D. middletonii*	BRIP 54884e	*Rapistrum rugostrum*	Australia	KJ197286	NA	NA	KJ197248	KJ197266
*D. miriciae*	BRIP 54736j	*Helianthus annuus*	Australia	KJ197282	NA	NA	KJ197244	KJ197262
*D. momicola*	MFLUCC 16-0113	*Prunus persica*	China	KU557563	KU557611	NA	KU557631	KU55758
*D. multigutullata*	ZJUD98	*Citrus grandis*	China	KJ490633	NA	KJ490575	KJ490512	KJ490454
***D. multigutullata***	**CFCC 53095**	***Citrus maxima***	**China**	**MK432645**	**MK442967**	**MK442992**	**MK578121**	**MK578048**
	**CFCC 53096**	***Citrus maxima***	**China**	**MK432646**	**MK442968**	**MK442993**	**MK578122**	**MK578049**
	**CFCC 53097**	***Citrus maxima***	**China**	**MK432647**	**MK442969**	**MK442994**	**MK578123**	**MK578050**
*D. musigena*	CBS 129519	*Musa* sp.	Australia	KC343143	KC343385	KC343627	KC343869	KC344111
*D. neilliae*	CBS 144.27	*Spiraea* sp.	USA	KC343144	KC343386	KC343628	KC343870	KC344112
*D. neoarctii*	CBS 109490	*Ambrosia trifida*	USA	KC343145	KC343387	KC343629	KC343871	KC344113
*D. oraccinii*	CGMCC 3.17531	*Camellia sinensis*	China	KP267863	NA	KP293517	KP267937	KP293443
*D. ovoicicola*	CGMCC 3.17093	*Citrus* sp.	China	KF576265	KF576223	NA	KF576240	KF576289
*D. pandanicola*	MFLU 18-0006	*Pandanus* sp.	Thailand	MG646974	NA	NA	NA	MG646930
*D. pascoei*	BRIP 54847	*Persea americana*	Australia	JX862532	NA	NA	JX862538	KF170924
*D. passifloricola*	CBS 141329	*Passiflora foetida*	Malaysia	KX228292	NA	KX228367	NA	KX228387
*D. penetriteum*	CGMCC 3.17532	*Camellia sinensis*	China	KP714505	NA	KP714493	KP714517	KP714529
*D. perjuncta*	CBS 109745	*Ulmus glabra*	Austria	KC343172	KC343414	KC343656	KC343898	KC344140
*D. perseae*	CBS 151.73	*Persea gratissima*	Netherlands	KC343173	KC343415	KC343657	KC343899	KC344141
*D. pescicola*	MFLUCC 16-0105	*Prunus persica*	China	KU557555	KU557603	NA	KU557623	KU557579
*D. podocarpi-macrophylli*	CGMCC 3.18281	*Podocarpus macrophyllus*	China	KX986774	KX999278	KX999246	KX999167	KX999207
*D. pseudomangiferae*	CBS 101339	*Mangifera indica*	Dominican Republic	KC343181	KC343423	KC343665	KC343907	KC344149
*D. pseudophoenicicola*	CBS 462.69	*Phoenix dactylifera*	Spain	KC343184	KC343426	KC343668	KC343910	KC344152
*D. psoraleae-pinnatae*	CBS 136413	*Psoralea pinnata*	South Africa	KF777159	NA	NA	NA	KF777252
*D. pterocarpicola*	MFLUCC 10-0580a	*Pterocarpus indicus*	Thailand	JQ619887	JX197433	NA	JX275403	JX275441
*D. pulla*	CBS 338.89	*Hedera helix*	Yugoslavia	KC343152	KC343394	KC343636	KC343878	KC344120
*D. pyracanthae*	CAA483	*Pyracantha coccinea*	Portugal	KY435635	KY435656	KY435645	KY435625	KY435666
*D. racemosae*	CBS 143770	*Euclea racemosa*	South Africa	MG600223	MG600219	MG600221	MG600225	MG600227
*D. rostrata*	CFCC 50062	*Juglans mandshurica*	China	KP208847	KP208849	KP208851	KP208853	KP208855
*D. sackstonii*	BRIP 54669b	*Helianthus annuus*	Australia	KJ197287	NA	NA	KJ197249	KJ197267
*D. sambucusii*	CFCC 51986	*Sambucus williamsii*	China	KY852495	KY852499	KY852503	KY852507	KY852511
***D. schimae***	**CFCC 53103**	***Schima superba***	**China**	**MK432640**	**MK442962**	**MK442987**	**MK578116**	**MK578043**
	**CFCC 53104**	***Schima superba***	**China**	**MK432641**	**MK442963**	**MK442988**	**MK578117**	**MK578044**
	**CFCC 53105**	***Schima superba***	**China**	**MK432642**	**MK442964**	**MK442989**	**MK578118**	**MK578045**
*D. schini*	CBS 133181	*Schinus terebinthifolius*	Brazil	KC343191	KC343433	KC343675	KC343917	KC344159
*D. schisandrae*	CFCC 51988	*Schisandra chinensis*	China	KY852497	KY852501	KY852505	KY852509	KY852513
*D. schoeni*	MFLU 15-1279	*Schoenus nigricans*	Italy	KY964226	KY964139	NA	KY964182	KY964109
*D. sennae*	CFCC 51636	*Senna bicapsularis*	China	KY203724	KY228875	NA	KY228885	KY228891
*D. serafiniae*	BRIP 55665a	*Helianthus annuus*	Australia	KJ197274	NA	NA	KJ197236	KJ197254
*D. siamensis*	MFLUCC 10-573a	*Dasymaschalon* sp.	Thailand	JQ619879	NA	NA	JX275393	JX275429
*D. sojae*	FAU635	*Glycine max*	USA	KJ590719	KJ612116	KJ659208	KJ590762	KJ610875
*D. sterilis*	CBS 136969	*Vaccinium corymbosum*	Italy	KJ160579	KJ160548	MF418350	KJ160611	KJ160528
*D. subclavata*	ICMP20663	*Citrus unshiu*	China	KJ490587	NA	KJ490529	KJ490466	KJ490408
*D. subellipicola*	MFLU 17-1197	Dead wood	China	MG746632	NA	NA	MG746633	MG746634
*D. subordinaria*	CBS 464.90	*Plantago lanceolata*	New Zealand	KC343214	KC343456	KC343698	KC343940	KC344182
*D. taoicola*	MFLUCC 16-0117	*Prunus persica*	China	KU557567	NA	NA	KU557635	KU557591
*D. tectonae*	MFLUCC 12-0777	*Tectona grandis*	China	KU712430	KU749345	NA	KU749359	KU743977
*D. tectonendophytica*	MFLUCC 13-0471	*Tectona grandis*	China	KU712439	KU749354	NA	KU749367	KU749354
*D. tectonigena*	MFLUCC 12-0767	*Tectona grandis*	China	KU712429	KU749358	NA	KU749371	KU743976
*D. terebinthifolii*	CBS 133180	*Schinus terebinthifolius*	Brazil	KC343216	KC343458	KC343700	KC343942	KC344184
*D. ternstroemia*	CGMCC 3.15183	*Ternstroemia gymnanthera*	China	KC153098	NA	NA	KC153089	NA
*D. thunbergii*	MFLUCC 10-576a	*Thunbergia laurifolia*	Thailand	JQ619893	JX197440	NA	JX275409	JX275449
*D. tibetensis*	CFCC 51999	*Juglandis regia*	China	MF279843	MF279888	MF279828	MF279858	MF279873
*D. tulliensis*	BRIP 62248a	*Theobroma cacao*	Australia	KR936130	NA	NA	KR936133	KR936132
*D. ukurunduensis*	CFCC 52592	*Acer ukurunduense*	China	MH121527	MH121445	MH121485	MH121569	NA
*D. unshiuensis*	CGMCC 3.17569	*Citrus unshiu*	China	KJ490587	NA	KJ490529	KJ490408	KJ490466
	CFCC 52594	*Carya illinoensis*	China	MH121529	MH121447	MH121487	MH121571	MH121606
*D. undulata*	CGMCC 3.18293	Leaf of unknown host	China-Laos border	KX986798	NA	KX999269	KX999190	KX999230
*D. vawdreyi*	BRIP 57887a	*Psidium guajava*	Australia	KR936126	NA	NA	KR936129	KR936128
***D. verniciicola***	**CFCC 53109**	***Vernicia montana***	**China**	**MK573944**	**MK574583**	**MK574599**	**MK574619**	**MK574639**
	**CFCC 53110**	***Vernicia montana***	**China**	**MK573945**	**MK574584**	**MK574600**	**MK574620**	**MK574640**
	**CFCC 53111**	***Vernicia montana***	**China**	**MK573946**	**MK574585**	**MK574601**	**MK574621**	**MK574641**
	**CFCC 53112**	***Vernicia montana***	**China**	**MK573947**	**MK574586**	**MK574602**	**MK574622**	**MK574642**
*D. viniferae*	JZB320071	*Vitis vinifera*	China	MK341551	MK500107		MK500119	MK500112
*D. virgiliae*	CMW40748	*Virgilia oroboides*	South Africa	KP247566	NA	NA	NA	KP247575
*D. xishuangbanica*	CGMCC 3.18282	*Camellia sinensis*	China	KX986783	NA	KX999255	KX999175	KX999216
***D. xunwuensis***	**CFCC 53085**	**Unknown dead wood**	**China**	**MK432663**	**MK442983**	**MK443008**	**MK578137**	**MK578063**
	**CFCC 53086**	**Unknown dead wood**	**China**	**MK432664**	**MK442984**	**MK443009**	**MK578138**	**MK578064**
*D. yunnanensis*	CGMCC 3.18289	*Coffea* sp.	China	KX986796	KX999290	KX999267	KX999188	KX999228
*Diaporthella corylina*	CBS 121124	*Corylus* sp.	China	KC343004	KC343246	KC343488	KC343730	KC343972

Newly sequenced material is indicated in bold type. NA, not applicable.

### Morphological observation

Agar plugs (6 mm diam.) were taken from the edge of actively-growing cultures on PDA and transferred on to the centre of 9 cm diam. Petri dishes containing 2% tap water agar, supplemented with sterile pine needles (PNA; Smith et al. 1996) and potato dextrose agar (PDA) and incubated at 25 °C under a 12 h near-ultraviolet light/12 h dark cycle to induce sporulation, as described in recent studies (Gomes et al. 2013; Lombard et al. 2014). Colony characters and pigment production on PNA and PDA were noted in the 10-day culture. Colony features were rated according to the colour charts of Rayner (1970). Cultures were examined periodically for the development of conidiomata. The microscopic examination was based on the morphological features of conidiomata obtained from the fungal growth, mounted in clear lactic acid. At least 30 conidia were measured to calculate the mean size/length. Micro-morphological observations were done at 1000× magnification using a Leica compound microscope (DM 2500) with interference contrast (DIC) optics. Descriptions, nomenclature and illustrations of taxonomic novelties were deposited at MycoBank (www.MycoBank.org).

### DNA extraction, PCR amplification and sequencing

Genomic DNA was extracted from colonies grown on cellophane-covered PDA, using a CTAB (cetyltrimethylammonium bromide) method (Doyle and Doyle 1990). DNA was estimated by electrophoresis in 1% agarose gel and the yield was measured using the NanoDrop 2000 (Thermo Scientific, Waltham, MA, USA), following the user manual (Desjardins et al. 2009). The PCR amplifications were performed in the DNA Engine Peltier Thermal Cycler (PTC-200; Bio-Rad Laboratories, Hercules, CA, USA). The primer set ITS1/ITS4 (White et al. 1990) was used to amplify the ITS region. The primer pair CAL228F/CAL737R (Carbone and Kohn 1999) was used to amplify the calmodulin gene (*cal*) and the primer pair CYLH4F (Crous et al. 2004) and H3-1b (Glass and Donaldson 1995) were used to amplify part of the histone H3 (*his3*) gene. The primer pair EF1-728F/EF1-986R (Carbone and Kohn 1999) was used to amplify a partial fragment of the translation elongation factor 1-α gene (*tef1*). The primer sets T1 (O’Donnell and Cigelnik 1997) and Bt2b (Glass and Donaldson 1995) were used to amplify the beta-tubulin gene (*tub2*); the additional combination of Bt2a/Bt2b (Glass and Donaldson 1995) was used in case of amplification failure of the T1/Bt2b primer pair. The PCR amplifications of the genomic DNA with the phylogenetic markers were done using the same primer pairs and conditions as in Yang et al. (2018). The PCR products were assayed via electrophoresis in 2% agarose gels, while the DNA sequencing was performed using an ABI PRISM 3730XL DNA Analyser with a BigDye Terminater Kit v.3.1 (Inv-itrogen, USA) at the Shanghai Invitrogen Biological Technology Company Limited (Beijing, China).

### Phylogenetic analyses

The quality of the amplified nucleotide sequences was checked and combined using SeqMan v.7.1.0 and reference sequences were retrieved from the National Center for Biotechnology Information (NCBI), based on recent publications on the genus *Diaporthe* (Guarnaccia et al. 2018; Yang et al. 2018, 2020). Sequences were aligned using MAFFT v. 6 (Katoh and Toh 2010) and corrected manually using Bioedit 7.0.9.0 (Hall 1999). The best-fit nucleotide substitution models for each gene were selected using jModelTest v. 2.1.7 (Darriba et al. 2012) under the Akaike Information Criterion.

The phylogenetic analyses of the combined gene regions were performed using Maximum Likelihood (ML) and Bayesian Inference (BI) methods. ML was conducted using PhyML v. 3.0 (Guindon et al. 2010), with 1000 bootstrap replicates while BI was performed using a Markov Chain Monte Carlo (MCMC) algorithm in MrBayes v. 3.0 (Ronquist et al. 2003). Two MCMC chains, started from random trees for 1,000,000 generations and trees, were sampled every 100^th^ generation, resulting in a total of 10,000 trees. The first 25% of trees were discarded as burn-in of each analysis. Branches with significant Bayesian Posterior Probabilities (BPP) were estimated in the remaining 7500 trees. Phylogenetic trees were viewed with FigTree v.1.3.1 (Rambaut and Drummond 2010) and processed by Adobe Illustrator CS5. Sequence alignment and phylogenetic trees were deposited in TreeBASE (submission ID: S25213). The nucleotide sequence data of the new taxa were deposited in GenBank (Table 1).

## Results

**Figure 1.**
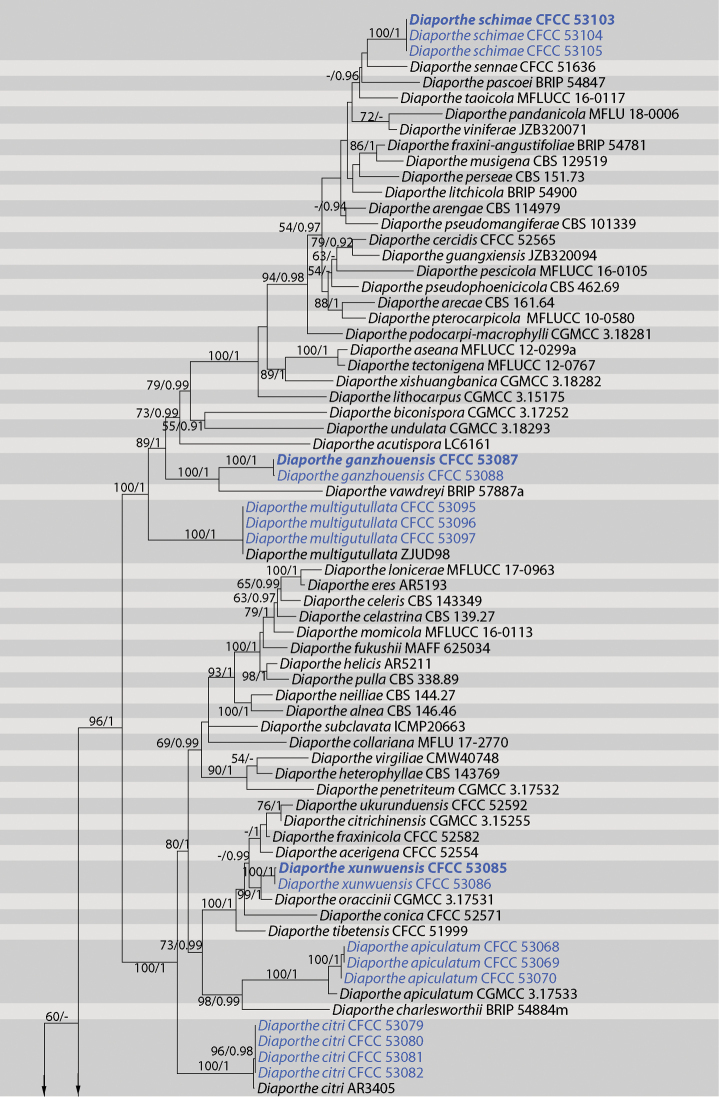
Phylogram of *Diaporthe* from a Maximum Likelihood analysis based on combined ITS, *cal*, *his3*, *tef1* and *tub2*. Values above the branches indicate Maximum Likelihood bootstrap (left, ML BP ≥ 50%) and Bayesian probabilities (right, BI PP ≥ 0.90). The tree is rooted with *Diaporthella corylina*. Strains in current study are in blue font and the ex-type cultures are in bold font.

The phylogenetic position of the 24 isolates of *Diaporthe* was determined by the phylogenetic analysis of the combined ITS, *cal*, *his3*, *tef1* and *tub2* sequences data. Reference sequences of the representative species used in the analysis were selected from Yang et al. (2018) and supplemented with sequences from GenBank. The ITS, *cal*, *his3*, *tef1tub2* and combined data matrices contained 522, 541, 529, 520, 535 and 2 659 characters with gaps, respectively. The alignment comprised of 142 strains together with *Diaporthella corylina* (culture CBS 121124) which was selected as the outgroup. The best nucleotide substitution model used for the analysis of ITS, *his3* and *tub2* was TrN+I+G, while HKY+I+G was used for *cal* and *tef1*. The topologies resulting from ML and BI analyses of the concatenated dataset were congruent (Fig. 1) and the sequences from the 24 *Diaporthe* isolates formed eight distinct clades as shown in Fig. 1, representing five undescribed species and three known species.

**Figure 11.**
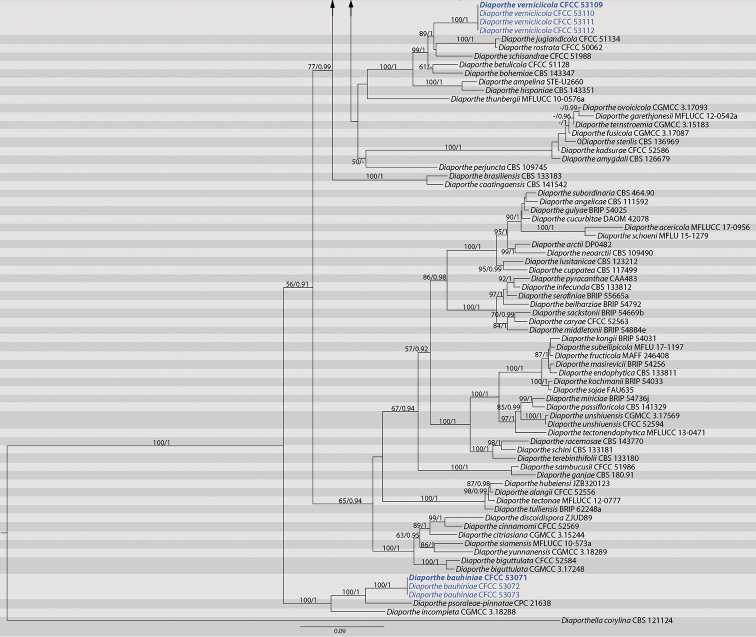
Continued.

### Taxonomy

#### Diaporthe apiculatum

Taxon classificationFungiDiaporthalesDiaporthaceae

Y.H. Gao & L. Cai, in Gao, Liu & Cai, Syst. Biodiv. 14: 106. 2016.

6FAC7E1B-E3D9-59EA-A41B-9780AD378F43

Figure 2

##### Description.

Conidiomata pycnidial, discoid, immersed in bark, scattered, slightly erumpent through bark surface, with a solitary undivided locule. Ectostromatic disc yellowish to grey, one ostiole per disc, (300–)305–357(–368) μm diam. Ostiole medium black, up to level of disc. Locule undivided, (338–)357–450(–464) μm diam. Conidiophores reduced to conidiogenous cells. Conidiogenous cells cylindrical, hyaline, densely aggregated, phiailidic, unbranched, straight or slightly curved. Beta conidia hyaline, aseptate, filiform, hamate, eguttulate, base subtruncate, tapering towards one apex, (26.5–)30–39.5(–43) × 1.5–2 µm. Alpha conidia not observed.

**Figure 2.**
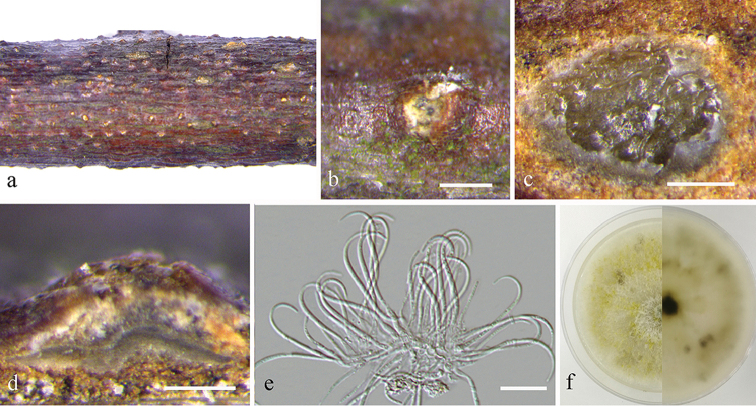
*Diaporthe apiculatum* on *Rhus chinensis* (BJFC-S1680) **a, b** habit of conidiomata in wood **c** transverse section of conidiomata **d** longitudinal section through conidiomata **e** conidiogenous cells attached with beta conidia **f** the colony on PDA. Scale bars: 200 μm (**b–d**); 10 μm (**e**).

##### Culture characters.

Colony originally flat with white fluffy aerial mycelium, becoming yellowish to pale green mycelium with age, marginal area irregular, conidiomata absent.

##### Specimens examined.

China. Jiangxi Province: Ganzhou City, Fengshan Forest Park, on branches of *Rhus chinensis*, 25°45'12"N, 115°00'41"E, 23 Jul 2018, *Q. Yang*, *Y. Liu*, *Y.M. Liang* & *C.M. Tian* (BJFC-S1680; living culture: CFCC 53068, CFCC 53069 and CFCC 53070).

##### Notes.

*Diaporthe apiculatum* was originally described as an endophyte from healthy leaves of *Camellia sinensis* in Jiangxi Province, China (Gao et al. 2015). In the present study, three isolates (CFCC 53068, CFCC 53069 and CFCC 53070) from symptomatic branches of *Rhus chinensis* were found congruent with *D. apiculatum*, based on DNA sequence and morphological data (Fig. 1). The clade was, therefore, confirmed to be *D. apiculatum* and was found to be both an endophyte and a pathogen.

#### Diaporthe bauhiniae

Taxon classificationFungiDiaporthalesDiaporthaceae

C.M. Tian & Q. Yang sp. nov.

13521E6A-F2E0-57FC-91D1-D6461FF110AC

829519

Figure 3

##### Diagnosis.

Distinguished from the phylogenetically closely-related species *D. psoraleae-pinnatae* in alpha and beta conidia.

##### Etymology.

Named after *Bauhinia*, the host genus where the fungus was isolated.

##### Description.

Conidiomata pycnidial, immersed in bark, scattered, slightly erumpent through bark surface, nearly flat, discoid, with a solitary undivided locule. Ectostromatic disc grey to brown, one ostiole per disc. Locule circular, undivided, (180–)200–290(–300) μm diam. Conidiophores reduced to conidiogenous cells. Conidiogenous cells hyaline, cylindrical, unbranched, straight, tapering towards the apex. Alpha conidia hyaline, aseptate, ellipsoidal to fusiform, biguttulate to multi-guttulate, (7.5–)9–13(–14) × (1.5–)2–2.5(–3) μm. Beta conidia hyaline, aseptate, filiform, straight to sinuous, eguttulate, (25–)28.5–40(–43) × 1 µm.

**Figure 3.**
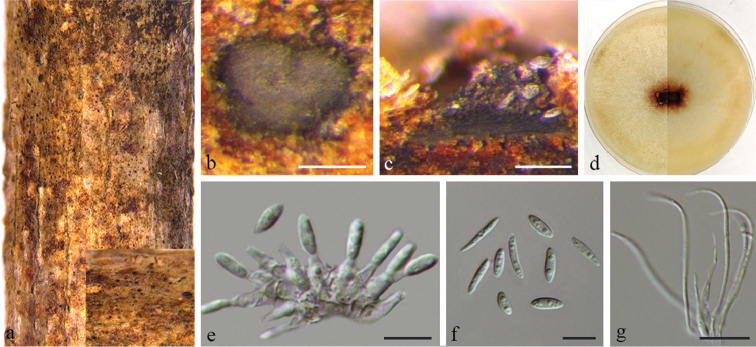
*Diaporthe bauhiniae* on *Bauhinia purpurea* (BJFC-S1621) **a** habit of conidiomata in wood **b** transverse section of conidiomata **c** longitudinal section through conidiomata **d** the colony on PDA**e** conidiogenous cells attached with alpha conidia **f** Alpha conidia **g** Beta conidia. Scale bars: 100 μm (**b, c**); 10 μm (**e–h**).

##### Culture characters.

Colony at first white, becoming wine-red in the centre with age. Aerial mycelium white, dense, fluffy, conidiomata absent.

##### Specimens examined.

China. Jiangxi Province: Ganzhou City, on branches of *Bauhinia purpurea*, 25°52'21"N, 114°56'44"E, 11 May 2018, *Q. Yang*, *Y. Liu* & *Y.M. Liang* (holotype BJFC-S1621; ex-type living culture: CFCC 53071; living culture: CFCC 53072 and CFCC 53073).

##### Notes.

Three isolates representing *D. bauhiniae* cluster in a well-supported clade and appear most closely related to *D. psoraleae-pinnatae*. *Diaporthe bauhiniae* can be distinguished from *D. psoraleae-pinnatae*, based on ITS and *tub2* (38/458 in ITS and 11/418 in *tub2*). Morphologically, *D. bauhiniae* differs from *D. psoraleae-pinnatae* in having narrower alpha conidia (2–2.5 vs. 2.5–3 μm) and the beta conidia of *D. psoraleae-pinnatae* were not observed (Crous et al. 2013).

#### Diaporthe citri

Taxon classificationFungiDiaporthalesDiaporthaceae

(H.S. Fawc.) F.A. Wolf, J. Agric. Res., Washington 33(7): 625, 1926.

6F1FD04B-82E2-56E3-AC79-428FF8679E08

Figure 4

##### Description.

Leaf spots subcircular to irregular, pale brown, with dark brown at margin. Pycnidia solitary, scattered on the leaf surface. Pycnidial conidiomata in culture, globose, erumpent, single or clustered in groups of 3–5 pycnidia, coated with hyphae, cream to yellowish translucent conidial droplets exuded from ostioles. Conidiophores reduced to conidiogenous cells. Conidiogenous cells hyaline, unbranched, septate, straight, slightly tapering towards the apex, 14.5–25 × 2–3 μm. Alpha conidia hyaline, aseptate, rounded at one end, apex at the other end, usually with two large guttulate, (9.5–)10.5–12 × 3.5–4.5 μm. Beta conidia not observed.

**Figure 4.**
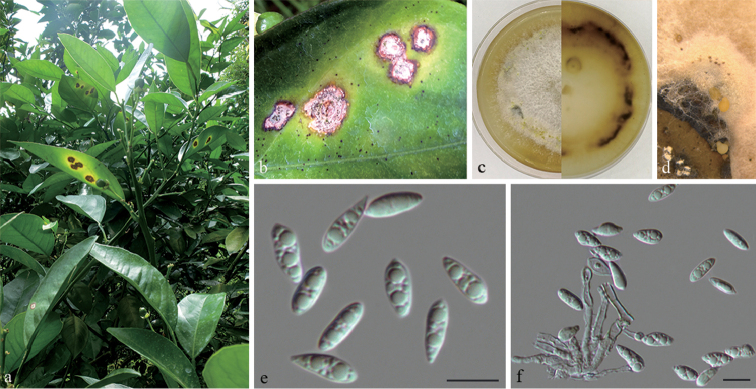
*Diaporthe citri* on *Citrus sinensis* (BJFC-S1658) **a, b** symptoms on leaves of host plant **c** culture on PDA (30d) **d** conidiomata **e** alpha conidia **f** conidiophores and alpha conidia. Scale bars: 10 μm (**e, f**).

##### Culture characters.

Colony originally flat with white fluffy aerial mycelium, becoming greyish mycelium with age, with yellowish-cream conidial drops exuding from the ostioles.

##### Specimens examined.

China. Jiangxi Province: Ganzhou City, on leaves of *Citrus sinensis*, 24°59'44"N, 115°31'01"E, 13 May 2018, *Q. Yang*, *Y. Liu* & *Y.M. Liang* (BJFC-S1658; living culture: CFCC 53079 and CFCC 53080); 24°59'45"N, 115°31'02"E, 13 May 2018, *Q. Yang*, *Y. Liu* & *Y.M. Liang* (BJFC-S1659; living culture: CFCC 53081 and CFCC 53082).

##### Notes.

*Diaporthe citri* is a widely distributed species in citrus-growing regions. In the present study, four isolates (CFCC 53079, CFCC 53080, CFCC 53081 and CFCC 53082) from symptomatic leaves of *Citrus sinensis* were congruent with *D. citri*, based on DNA sequence and morphological data (Fig. 1). The clade was, therefore, confirmed to be *D. citri*.

#### Diaporthe ganzhouensis

Taxon classificationFungiDiaporthalesDiaporthaceae

C.M. Tian & Q. Yang sp. nov.

90B3BE1F-2BF5-5865-BCDC-C58684BB6AEE

829522

Figure 5

##### Diagnosis.

Distinguished from the phylogenetically closely-related species *D. vawdreyi* in having longer conidiophores and wider alpha conidia.

##### Etymology.

Named after Ganzhou City where the species was first collected.

##### Description.

On PDA: Conidiomata pycnidial, subglobose, solitary, deeply embedded in the medium, erumpent, dark brown to black. Pale yellow conidial drops exuding from ostioles. Conidiophores (12–)15.5–21 × 1.5–2 μm, cylindrical, hyaline, phiailidic, branched, straight or slightly curved. Alpha conidia 6.5–8.5(–9) × 2–2.5(–3) μm, aseptate, hyaline, ellipsoidal to fusiform, rounded at one end, slightly apex at the other end, biguttulate. Beta conidia hyaline, aseptate, filiform, sinuous at one end, eguttulate, (21.5–)25.5–31(–33) × 1 µm.

**Figure 5.**
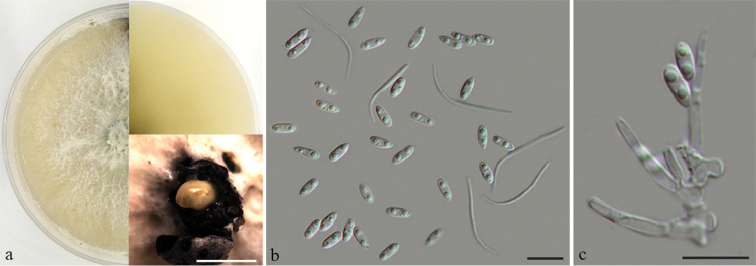
*Diaporthe ganzhouensis* on unknown host (BJFC-S1678) **a** the colony on PDA and conidiomata **b** alpha and beta conidia **c** conidiogenous cells and alpha conidia. Scale bars: 10 μm (**b, c**).

##### Culture characters.

Colony at first white, becoming yellowish with age. Aerial mycelium white, dense, fluffy, with visible solitary conidiomata at maturity.

##### Specimens examined.

China. Jiangxi Province: Ganzhou City, unknown dead wood, 25°45'17"N, 115°00'41"E, 23 Jul 2018, *Q. Yang*, *Y. Liu*, *Y.M. Liang* & *C.M. Tian* (holotype BJFC-C004; ex-type culture: CFCC 53087; living culture: CFCC 53088).

##### Notes.

*Diaporthe ganzhouensis* comprises the isolates CFCC 53087 and CFCC 53088, revealed to be closely related to *D. vawdreyi* in the combined phylogenetic tree (Fig. 1). *Diaporthe ganzhouensis* can be distinguished, based on ITS, *tef1-α* and *tub2* loci from *D. vawdreyi* (6/456 in ITS, 63/357 in *tef1-α* and 40/469 in *tub2*). *Diaporthe ganzhouensis* differs morphologically from *D. vawdreyi* in having longer conidiopores (15.5–21 vs. 6–15 μm) and wider alpha conidia (2–2.5 vs. 1.5–2 μm) (Crous et al. 2015).

#### Diaporthe multiguttulata

Taxon classificationFungiDiaporthalesDiaporthaceae

F. Huang, K.D. Hyde & Hong Y. Li, in Huang et al., Fungal Biology 119(5): 343. 2015.

FF202454-C6E7-5DAF-AFA6-F783DE9AA27A

Figure 6

##### Description.

Conidiomata pycnidial, 692–750(–800) μm diam., solitary and with single necks erumpent through host bark. Tissue around neck is cylindrical. Locule circular, undivided, 450–565(–600) μm diam. Conidiophores reduced to conidiogenous cells. Conidiogenous cells unbranched, straight or slightly curved, apical or base sometimes swelling, (8.5–)9–10.5(–11) × 1.5–2 μm. Alpha conidia hyaline, aseptate, ellipsoidal, biguttulate or with one large guttulate, rounded at one end, slightly apex at the other end, occasionally submedian constriction, (7.5–)8–9(–10.5) × 4–5(–5.5) μm. Beta conidia not observed.

**Figure 6.**
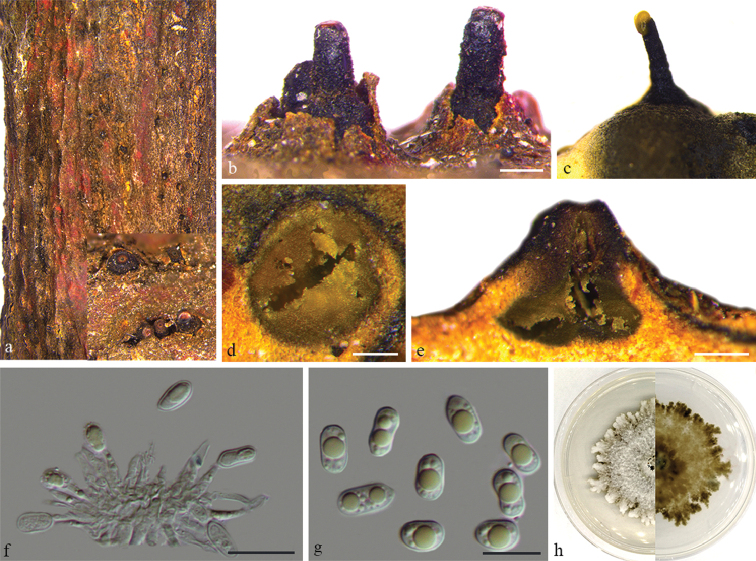
*Diaporthe multiguttulata* on *Citrus maxima* (BJFC-S1614) **a, b** habit of conidiomata on twig **c** conidiomata on PDA**d** transverse section through conidiomata **e** longitudinal section through conidiomata **f** conidiogenous cells attached with alpha conidia **g** alpha conidia **h** the colony on PDA. Scale bars: 200 μm (**b, d, e**); 10 μm (**f, g**).

##### Culture characters.

Colony originally flat with white felty aerial mycelium, becoming pale green mycelium with age, margin area irregularly, with visible solitary conidiomata at maturity.

##### Specimens examined.

China. Jiangxi Province: Ganzhou City, on branches of *Citrus maxima*, 25°51'28"N, 114°55'19"E, 11 May 2018, *Q. Yang*, *Y. Liu* & *Y.M. Liang* (BJFC-S1614; living culture: CFCC 53095, CFCC 53096 and CFCC 53097).

##### Notes.

*Diaporthe multiguttulata* was originally described as an endophyte from a healthy branch of *Citrus grandis* in Fujian Province, China (Huang et al. 2015). In the present study, three isolates (CFCC 53095, CFCC 53096 and CFCC 53097) from symptomatic branches of *Citrus maxima* were congruent with *D. multigutullata*, based on DNA sequence data and confirmed from the morphological analysis (Fig. 1). The clade, therefore, was verified as *D. multigutullata* which could exist both as an endophyte and a pathogen.

#### Diaporthe schimae

Taxon classificationFungiDiaporthalesDiaporthaceae

C.M. Tian & Q. Yang sp. nov.

B56097C9-3E21-5805-B9F2-80D8060CA4A3

829526

Figure 7

##### Diagnosis.

Distinguished from the phylogenetically closely-related species *D. sennae* in having larger alpha conidia and longer beta conidia.

##### Etymology.

Named after the host genus *Schima* on which the fungus was isolated.

##### Description.

Leaf spots subcircular to irregular, pale brown, with dark brown at margin. Pycnidia solitary, scattered on the leaf surface. Pycnidial conidiomata in culture, globose, (150–)173–357(–373) µm in its widest diam., erumpent, single or clustered in groups of 3–5 pycnidia, coated with hyphae, cream to yellowish translucent conidial droplets exuded from ostioles. Conidiophores reduced to conidiogenous cells. Conidiogenous cells hyaline, unbranched, septate, straight, slightly tapering towards the apex. Alpha conidia scarce, hyaline, aseptate, ellipsoidal to spindle-shaped, four small guttulate, (7.5–)8–8.5(–9) × 2.5–3 μm. Beta conidia abundant, hyaline, aseptate, filiform, straight to sinuous at one end, eguttulate, (25–)27.5–38.5(–40.5) × 1–1.5 µm.

**Figure 7.**
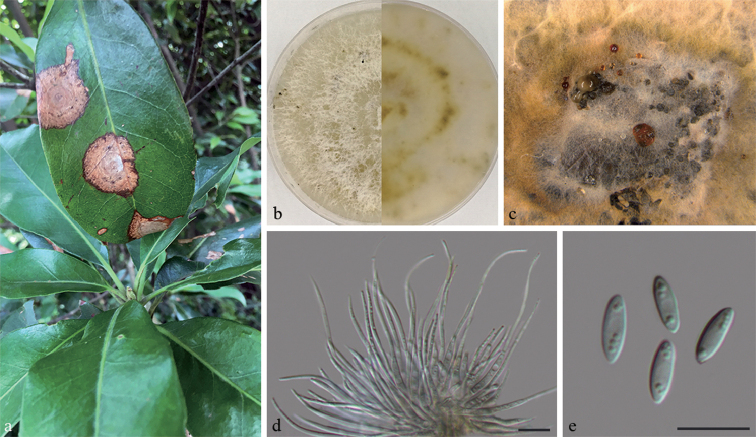
*Diaporthe schimae* on *Schima superba* (BJFC-S1661) **a** symptoms on leaves of host plant **b** the colony on PDA**c** conidiomata on PDA**d** conidiophores cells attached with beta conidia **e** Alpha conidia. Scale bars: 10 μm (**d, e**).

##### Culture characters.

Colony entirely white, with fluffy aerial mycelium, concentric zonation, margin fimbricate, reverse slightly yellowish.

##### Specimens examined.

China. Jiangxi Province: Ganzhou City, Fengshan Forest Park, on leaves of *Schima superba*, 25°44'22"N, 114°59'40"E, 15 May 2018, *Q. Yang*, *Y. Liu* & *Y.M. Liang* (holotype BJFC-S1661; ex-type culture: CFCC 53103); 24°40'51"N, 115°34'36"E, 15 May 2018, *Q. Yang*, *Y. Liu* & *Y.M. Liang* (BJFC-S1662; living culture: CFCC 53104); 24°40'52"N, 115°34'54"E, 15 May 2018, *Q. Yang*, *Y. Liu* & *Y.M. Liang* (BJFC-S1663; living culture: CFCC 53105).

##### Notes.

*Diaporthe schimae* occurs in an independent clade (Fig. 1) and was revealed to be phylogenetically distinct from *D. sennae*. *Diaporhe schimae* can be distinguished with *D. sennae* by 41 nucleotides in concatenated alignment, in which three were distinct in the ITS region, 20 in the *tef1-α* region and 18 in the *tub2* region. *Diaporthe schimae* differs morphologically from *D. sennae* in having larger alpha conidia and longer beta conidia (8–8.5 × 2.5–3 vs. 5.5–6.3 × 1.5–1.7 µm in alpha conidia; 27.5–38.5 vs. 18.4–20 µm in beta conidia) (Yang et al. 2017a).

#### Diaporthe verniciicola

Taxon classificationFungiDiaporthalesDiaporthaceae

C.M. Tian & Q. Yang sp. nov.

F4EAE0F5-46B8-58C0-B937-A95F82DCDF39

832921

Figure 8

##### Diagnosis.

Distinguished from the phylogenetically closely-related species *D. rostrata* in having smaller alpha conidia; and from *D. juglandicola* in having wider alpha conidia.

##### Etymology.

Named after the host genus *Vernicia* on which the fungus was isolated.

##### Description.

Conidiomata pycnidial, 825–1050 × 445–500 μm diam., solitary and with single necks erumpent through host bark. Tissue around neck is conical. Locule circular, undivided, 400–665 μm diam. Conidiophores reduced to conidiogenous cells. Conidiogenous cells unbranched, straight or sinuous, 14.5–21.5 × 1–1.5 μm. Alpha conidia hyaline, aseptate, ellipsoidal to fusiform, with 1–2-guttulate, 7–8.5 × 3–3.5 μm. Beta conidia not observed.

**Figure 8.**
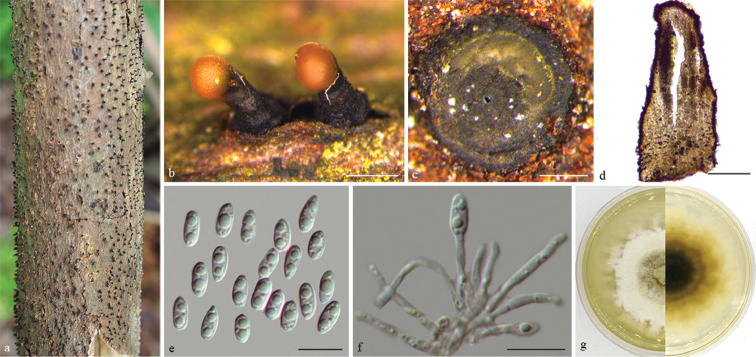
*Diaporthe verniciicola* on *Vernicia montana* (BJFC-S1622) **a, b** habit of conidiomata on twig **c** transverse section through conidiomata **d** longitudinal section through conidiomata **e** alpha conidia **f** conidiophores **g** culture on PDA (30d). Scale bars: 500 μm (**b**); 200 μm (**c**); 10 μm (**e, f**).

##### Culture characters.

Colony white to yellowish, with dense and felted mycelium in the centre, lacking aerial mycelium, conidiomata absent.

##### Specimens examined.

China. Jiangxi Province: Ganzhou City, on branches of *Vernicia montana*, 24°40'51"N, 115°34'52"E, 12 May 2018, *Q. Yang*, *Y. Liu* & *Y.M. Liang* (holotype BJFC-S1622; ex-type culture: CFCC 53109); 24°40'52"N, 115°34'50"E, 12 May 2018, *Q. Yang*, *Y. Liu* & *Y.M. Liang* (BJFC-S1623; living culture: CFCC 53110); 24°45'14"N, 115°34'00"E, 12 May 2018, *Q. Yang*, *Y. Liu* & *Y.M. Liang* (BJFC-S1624; living culture: CFCC 53111); 25°44'15"N, 114°59'32"E, 15 May 2018, *Q. Yang*, *Y. Liu* & *Y.M. Liang* (BJFC-S1624; living culture: CFCC 53112).

##### Notes.

Two isolates of *D. verniciicola* clustered in a well-supported clade (ML/BI = 100/1) and appeared closely related to *D. rostrata* and *D. juglandicola* (Fig. 1). Morphologically, *D. verniciicola* is similar to *D. rostrata* characterised by conidiomata with single necks erumpent through the host bark. However, the new taxon can be distinguished from *D. rostrata* in having smaller alpha conidia (7–8.5 × 3–3.5 vs. 8.5–11.5 × 4–5 μm) (Fan et al. 2015) and *D. verniciicola* differs from *D. juglandicola* in having wider alpha conidia (3–3.5 vs. 2.5–3 μm) (Yang et al. 2017b). This is the first discovery of a *Diaporthe* species isolated from infected branches or twigs on *Vernicia montana* and was confirmed as a new species, based on phylogeny and morphology.

#### Diaporthe xunwuensis

Taxon classificationFungiDiaporthalesDiaporthaceae

C.M. Tian & Q. Yang sp. nov.

6EF44D7F-DB53-53B3-99E0-B6C9247F20A0

829521

Figure 9

##### Diagnosis.

Distinguished from the phylogenetically closely-related species *D. oraccinii* in having longer conidiophores and larger alpha conidia.

##### Etymology.

Named after the county (Xunwu) where the species was first collected.

##### Description.

On PDA: Conidiomata pycnidial, globose, solitary or aggregated, deeply embedded in the medium, erumpent, dark brown to black. Hyaline conidial drops exuding from ostioles. Conidiophores (18.5–)21.5–30(–32.5) × 1–1.5(–2) μm, cylindrical, hyaline, phiailidic, unbranched, straight to sinuous. Alpha conidia (6.5–)7–8.5 × 2–3 μm, aseptate, hyaline, ellipsoidal to fusiform, rounded at one end, slightly apex at the other end, usually with 2-guttulate. Beta conidia not observed.

**Figure 9.**
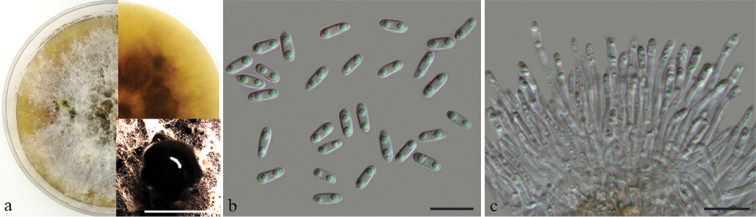
*Diaporthe xunwuensis* on unknown host (BJFC-S1679) **a** the colony on PDA and conidiomata **b** alpha conidia **c** conidiogenous cells attached with alpha conidia. Scale bars: 10 μm (**a–c**).

##### Culture characters.

Colony at first white, becoming dark brown in the centre with age. Aerial mycelium white, dense, fluffy, with black conidial drops exuding from the ostioles.

##### Specimens examined.

China. Jiangxi Province: Ganzhou City, unknown dead wood, 25°45'17"N, 115°00'41"E, 23 Jul 2018, *Q. Yang*, *Y. Liu*, *Y.M. Liang* & *C.M. Tian* (holotype BJFC-C003; ex-type culture: CFCC 53085; living culture: CFCC 53086).

##### Notes.

Two isolates representing *D. xunwuensis* clustered in a well-supported clade and appear most closely related to *D. oraccinii*. *Diaporthe xunwuensis* can be distinguished from *D. oraccinii*, based on ITS, *his3* and *tef1-α* loci (5/471 in ITS, 5/432 in *his3* and 5/325 in *tef1-α*). Morphologically, *D. xunwuensis* differs from *D. oraccinii* in having longer conidiopores (21.5–30 vs. 10.5–22.5 μm) and larger alpha conidia (7–8.5 × 2–3 vs. 5.5–7.5 × 0.5–2 μm) (Gao et al. 2016).

## Discussion

The current study described eight *Diaporthe* species from 24 strains, based on a large set of freshly-collected specimens. It includes five new species and three known species, which were sampled from six host genera distributed in Jiangxi Province of China (Table 1). In this study, 142 reference sequences (including outgroup) were selected, based on BLAST searches of NCBIs GenBank nucleotide database and included in the phylogenetic analyses (Table 1). Phylogenetic analyses, based on five combined loci (ITS, *cal*, *his3*, *tef1* and *tub2*), as well as morphological characters, revealed the diversity of *Diaporthe* species in Jiangxi Province, mainly focusing on diebacks from major ecological or economic forest trees.

The identification and characterisation of novel taxa and new host records indicate the high potential of *Diaporthe* to evolve rapidly. In the present study, five species were first reported in China as pathogens. Amongst these species, *D. bauhiniae* was characterised by having longer alpha conidia (9–13 × 2–2.5 μm). *Diaporthe ganzhouensis* and *D. xunwuensis* were isolated from unknown dead wood, but *D. ganzhouensis* can be distinguish from *D. xunwuenesis* in having beta conidia and was supported by analysis of the sequence data. *Diaporthe schimae* was identified as the most widespread species from isolates collected in Jiangxi Province. *Diaporthe verniciicola* have conidiomata with single necks erumpent through the host bark. Furthermore, two new host records were described, *D. apiculatum* from *Rhus chinensis* and *D. multiguttulata* from *Citrus maxima*.

Recent plant pathological studies have revealed that several *Diaporthe* species cause disease, particularly to important plant hosts on a wide range of economically-significant agricultural crops, such as blueberries, citrus, grapes, oaks, sunflowers, soybeans, tea plants, tropical fruits, vegetables and various trees (van Rensburg et al. 2006; Santos and Phillips 2009; Santos et al. 2011; Thompson et al. 2011; Grasso et al. 2012; Lombard et al. 2014; Huang et al. 2015; Udayanga et al. 2015; Gao et al. 2016; Guarnaccia et al. 2018; Yang et al. 2020). For example, research conducted by Huang et al. (2015) revealed seven endophytic *Diaporthe* species on *Citrus*; Gao et al. (2016) demonstrated that *Diaporthe* isolates associated with *Camellia* spp. could be assigned to seven species and two species complexes; Guarnaccia et al. (2018) explored the occurrence, diversity and pathogenicity of *Diaporthe* species associated with *Vitis vinifera* and revealed four new *Diaporthe* species; Yang et al. (2018) provided the first molecular phylogenetic framework of *Diaporthe* diversity associated with dieback diseases in China. Following the adoption of DNA sequence-based methods, *Diaporthe* taxonomy is actively changing, with numerous species being described each year.

The present study is the first evaluation of *Diaporthe* species, associated with dieback diseases in Jiangxi Province using the combined morphology and molecular data and provided useful information for evaluating the pathogenicity of various species. Multiple strains from different locations should also be subjected to multi-locus phylogenetic analysis to determine intraspecific variation and redefine species boundaries. The descriptions and molecular data of *Diaporthe* species, provided in this study, represent a resource for plant pathologists, plant quarantine officials and taxonomists for identification of *Diaporthe*.

## Supplementary Material

XML Treatment for Diaporthe apiculatum

XML Treatment for Diaporthe bauhiniae

XML Treatment for Diaporthe citri

XML Treatment for Diaporthe ganzhouensis

XML Treatment for Diaporthe multiguttulata

XML Treatment for Diaporthe schimae

XML Treatment for Diaporthe verniciicola

XML Treatment for Diaporthe xunwuensis
